# Evaluating the success of Slovenia’s policy on the health of children and adolescents: results of an audit

**DOI:** 10.1007/s00038-020-01432-0

**Published:** 2020-07-17

**Authors:** Tine Bizjak, Rok Novak, Marko Vudrag, Andreja Kukec, Branko Kontić

**Affiliations:** 1 Department of Environmental Sciences, Jožef Stefan Institute, Ljubljana, Slovenia; 2 Jožef Stefan International Postgraduate School, Ljubljana, Slovenia; 3 National Institute of Public Health, Ljubljana, Slovenia; 4 Centre of Public Health, Faculty of Medicine, University of Ljubljana, Ljubljana, Slovenia; 5 Department for Environmental Health, National Institute of Public Health, Ljubljana, Slovenia

**Keywords:** Public health, Adolescent health, Auditing, Environmental quality, Indicators

## Abstract

**Objectives:**

The aims of this audit were twofold: (1) to demonstrate the contribution of the auditing process in evaluating the success of child and adolescent health policy in Slovenia between 2012 and 2019, and (2) to expand on the commentary published in the International Journal of Public Health in 2019 to demonstrate the benefits of auditing in improving public health policy in general.

**Methods:**

The audit followed health, safety and environmental approaches as per the standards of public health policy.

**Results:**

Due to poor intersectoral coordination and weak associations between environmental and health indicators, no clear evidence could be established that child and adolescent health policy contributed to positive changes in child and adolescent health from 2012 to 2019.

**Conclusions:**

Auditing should become an essential component of measuring the success of public health policies. Attention should also be paid to the following issues affecting youth health: sleeping and eating habits, economic migration, poverty, etc.

## Introduction

In a piece of 2019 commentary published in the International Journal of Public Health, ‘Auditing in addition to compliance monitoring: a way to improve public health’, authors stressed that the actual effects of public health policy on society is determined by the quality of its implementation (Bizjak and Kontić 2019). They further argued a key condition for ensuring health policies’ successful implementation: an active system of responsible and competent authorities capable of prioritizing issues, assigning responsibilities and effectively distributing the available budget. Such a system invariably entails continuous monitoring to evaluate the success of implemented measures, assess the extent to which goals are achieved and identify barriers in attempted policy improvements. In terms of monitoring policy implementation, there are some caveats regarding limited information about its performance (Kaur 2010; Usmanova and Mokdad 2013; van den Driessen Mareeuw et al. 2015; Donkor et al. 2018; Gulis 2019). In this context, the European Commission recently stressed the importance of learning from assessments of existing air quality legislation in view of regularly updating public health policy (The Green Deal; European Commission 2019). However, despite general recognition that auditing is beneficial, few studies focus on the effectiveness of public health or health services (Kingdon 1995; Brownson et al. 2010; Shankar et al. 2011; Singh 2014; Bradley et al. 2016; Bernet et al. 2018).

To demonstrate that auditing is an effective tool in identifying possibilities to improve public health in Slovenia, an agreement was made in 2019 between national public health professionals and an auditing team to check the performance of the national strategy on children and adolescent health related to environmental quality for the period 2012–2020 (referred to as the Strategy, the Government of the Republic of Slovenia 2011; see summary below). This Strategy was selected for the following characteristics: (1) it is a national level policy; (2) it builds on international efforts and policies regarding health and environmental initiatives [World Health Organisation (WHO), United Nations Environment Programme, United Nations Development Programme, European Environment Agency, European Food and Safety Agency, etc.]; (3) it is accompanied by a specific action plan to implement the Strategy (referred to as the action plan (AP); Government of the Republic of Slovenia 2015), which details priority goals, related activities, monitoring indicators, etc.; and (4) there is an intergovernmental working group (IWG) that has been established to follow the implementation of the Strategy and regularly report its findings to the government. The audit lasted from September 2019 to April 2020 with an open end for a post-audit phase. This was occasioned by the changed priorities triggered by the COVID-19 pandemic. The scope and foci of the audit are depicted in Table 1.

**Table 1:** Auditing topics and relations (applicable generally when auditing public health policies)

Auditing topics and relations	Rationale
1. Character of the Strategy: preventive/curative/both	Differences between the preventive and curative character of the Strategy can direct the auditing process towards either (1) examining whether the Strategy’s success should be evaluated in terms of fixing pressing issues, leading to improved future circumstances (curative) or (2) examining the Strategy’s success in preventing public health status from worsening compared to the outset of the Strategy’s implementation (preventive)
2. Consistency between the Strategy and the AP: substance, timing, activities, responsible bodies and indicators	The audit checks whether the Strategy and its AP accord with one another and are complete. If so, the credibility and trustworthiness of both can be confirmed; otherwise, inconsistent or/and conflicting issues should be identified and fixed prior to any barriers to implementation
3. Functional strength of the indicators: clearness, measurability, meaning and associations and history of record	Indicators should be ‘fit for purpose’. This means that they provide information as needed, allowing for tractable intermediate and final examinations of the Strategy’s success
4. Links between environmental quality and health indicators	The Strategy deals with adolescent health in relation to environmental quality. This is the core of the overall evaluation of the measures applied through the Strategy and the AP. Indicators applied throughout the Strategy’s implementation should be accordingly selected and synthesised
5. Evaluation of association, possibly causality, between environmental quality and health status changes as determined by the indicators’ values	Similar to the one given in pt. 4; if causality is to be established, proper evidence-based information—e.g. measures commonly applied in epidemiology—should support interpretation of indicators’ values pertaining to the evaluation period
6. Strategy compliance with legal and agreed-upon commitments	Compliance is a standard component of auditing
7. Expected versus actual work of the IWG: accountability, transparency, intervention (as needed), meeting frequency, coordination, management and recording and reporting	Management performance is a key auditing component. It contributes to the Strategy’s overall credibility and trustworthiness. Responsible behaviour is one of the related topics
8. Evaluation of the Strategy’s success: children and adolescent health status improvement during the period 2012–2020, proposals for future work	The audit checks whether this final step of the Strategy implementation has been conducted comprehensively and as per the prescribed quality standards
9. Overall transparency and participation of interested parties	The audit assesses the democratic aspects of the Strategy

*AP* action plan, *IWG* intergovernmental working group

### Summary of the strategy

By signing the Parma Declaration in 2010 (WHO Regional Office for Europe 2010), the Republic of Slovenia has committed itself to protecting adolescent health against harmful environmental factors, acknowledging it as an integral part of the country’s public health and environmental policies. Other important backgrounds of the Strategy are the European Environment and Health Strategy (EC 2003), European Environment and Health Action Plan 2004–2010 (EC 2004) and the 6th Environment Action Programme of the European Community 2002–2012 (EC 2011).

On 29 July 2010, the Slovenian government appointed the IWG to implement the commitments of the Strategy. The IWG’s first task was preparing the Adolescent Environmental Health Action Programme and the Chemical Safety Action Programme, which were merged to form the Strategy.

The Strategy determined four general priority goals: (1) ensuring population health by improving access to safe drinking water and appropriate municipal wastewater management, (2) reducing injury and obesity through safe environments and healthy diet paired with physical activity, respectively, (3) preventing disease by improving indoor and outdoor air quality and (4) preventing diseases caused by chemical, biological and physical risk factors. The AP further specified the activities leading to the achievement of goals, the duration of said activities, monitoring indicators and the institutions responsible. Specific areas of focus were also determined, such as youth participation, climate change, inequality, new technology and excessively polluted areas.

The WHO/ENHIS indicators, combined with those developed by the National Institute of Public Health (NIJZ) and Slovenian Environment Agency, were applied in the context of monitoring the effects of Strategy implementation. The initial set included regulatory aspects of environmental protection, air pollution in cities, drinking water quality, infant mortality due to respiratory disease, asthma and allergic diseases in children, child exposure to polluted air—PM_10_ particles, waterborne disease outbreaks (epidemics), access to safe drinking water, etc. The indicators had to be updated regularly to properly capture new and additional views on the relationships between exposure to environmental risk factors and observed health outcomes. Some additional health indicators were obesity, diabetes, congenital irregularities, etc. Annual surveys and reporting of adolescent health status according to these indicators were to be provided by the NIJZ.

The Strategy defined that the IWG will report to the Government every 2 years on the Strategy’s implementation progress, the findings of which would be used to plan future health and environmental policy.

## Methods

**Fig. 1:**
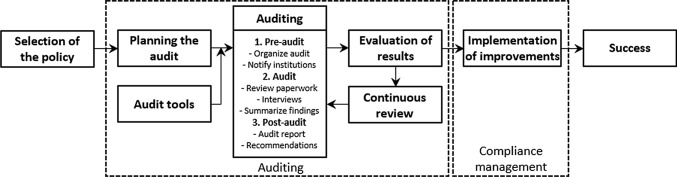
Elements of the audit programme

The key aspects and principles of auditing were applied according to the definitions and guidance offered by Cahill et al. (1987), INTOSAI (2004), CCPS (2011) and ECA (2017). Adaptations to the area of public health policy followed the experience of Brownson et al. (2010), Shankar et al. (2011) and Bernet et al. (2018). Figure 1 shows the main elements of an established audit programme. Standard auditing tools, such as questionnaires, worksheets, guidelines, etc. were used to collect, sort, analyse and retrieve audit information.

The audit was based on reviewing the Strategy’s AP and the annual reporting of environmental quality and related health status from 2012 to 2019, provided by the NIJZ. Interviewing the personnel engaged in Slovenian public health policy preparation, primarily from the Ministry of Health, the NIJZ and the Ministry of the Environment and Spatial Planning, was performed to verify specific policy information in the reviewed documents in the context of intersectoral coordination. The head of the IWG was also consulted regarding its work.

In the pre-audit phase, three meetings were held with experts from the three institutions engaged in preparing the Strategy. At these meetings, which were also associated with work on the European Union-funded project on the Health and Environment Research Agenda (HERA: https://www.heraresearcheu.eu/) for Europe, the selection of documents for review were discussed and approved. Since the initially selected documentation covered practically all components of environmental and public health issues, the audit team decided to narrow the scope and perform the audit only for the Strategy documents. The key reasons for this relate to the characteristics of the Strategy as described in the introduction in items (1) through (4).

The evaluation was conducted to compare the health status of children and adolescents before and after the Strategy’s implementation. The attempt was to assign (positive) changes to the Strategy and related AP activities. Key metrics were based on associations between selected health and environmental indicators, and trends in the observed period were to be analysed. The overall policy evaluation included the following topics: design and consistency between the Strategy and AP, implementation monitoring, outcome variables (i.e. the performance of the activities and their results: qualitative, qualitative or both), transparency and reporting and availability of data for evaluation. Some indicators were quantitative (e.g. share of monitored drinking water and measured air quality parameters), while others required combined quantitative and qualitative metrics (e.g. determining if and to what extent municipalities follow public health guidelines). The evaluation categories, applied in Tables 2 and 3, were:

**Table 2:** Audit findings for priority Goal 1 (Slovenia 2012–2019)

Priority Goal 1: Ensuring population health by improving access to safe drinking water and appropriate municipal wastewater management			
Activities planned for achieving the expected results—AP Implementation of the Protocol on Water and Health. Activity 1 Improved access to safe drinking water, municipal wastewater management. Activities 2–7 Quality control of swimming water. Activities 8 and 9			
AP activities	Indicators^a^ and performance evaluation of activities	Audit findings	
		Consistency	Additional comments (according to Table 1)
1. Protocol on Water and Health	Ratification of the Protocol Status/Score: W	Y	Protocol prepared but not ratified
2. Water protection areas. Raising awareness about conservation of drinking water resources	2.1 Share of protected water resources Status/Score: O 2.2. Awareness-raising about the importance of good quality/safe drinking water through nature conservation Status/Score: X 2.3 Population with unknown drinking water quality (APR) Status/Score: W 2.4 Microbiologically non-compliant drinking water samples (APR) Status/Score: G 2.5 Exposure to nitrates and pesticides in drinking water (APR) Status/Score: O	P (e.g. GIS supported monitoring system)	2.1 Data not available; water protection areas remained unchanged during 2013–2016 2.2 Indicator not auditable 2.3 Share of the population whose drinking water resources were not monitored was reduced from 7.3% in 2012 to 5.8% in 2018 2.4 Share of microbiologically non-compliant drinking water dropped from 16% in 2012 to 12% in 2018 2.5 No trends observed. Number of exposed varies. Data on drinking water quality and infectious diseases cannot be clearly associated. Annual data on water quality is not comparable (different sampling)
3. Connectivity of relevant databases	Connectivity of databases Status/Score: X	N (activities introduced by the NIJZ in 2016)	Indicator not auditable: no data
4. Measures for safe and economical use of drinking water facilities	4.1 Number of actions Status/Score: X 4.2 Number of waterborne infection outbreaks Status/Score: W	Y	4.1 Indicator not auditable: no data 4.2 Only a few outbreaks were reported between 2012 and 2017, and the number of infected was below 100 except in 2016 (around 400). About 60% of gastroenterocolitis cases were of unknown etiology
5. Treatment of municipal wastewater	5.1 Proportion of treated wastewater Status/Score: G 5.2 Number of gastroenterocolitis cases in children and youth under 15 years of age (APR) Status/Score: W	P (e.g. no clear goals set)	5.1 Share of population with treated wastewater increased by about 20%, share of tertiary treatment by about 25% (2012–2018) 5.2 No trend observed. The 1–4 year and 5–14 year age groups have consistently had the highest infections rates (e.g. 7206 and 5891 out of 29,168 cases in 2015, respectively; 2632 and 3510 out of 10,493 cases in 2018, respectively). The majority of cases were of unknown etiology
6. Hygiene practices of vulnerable groups	Actions taken in this area Status/Score: X	N	Indicator not auditable Limited effect of the national programme on Roma is reported (Okorn 2016)
7. Raising awareness about the importance of good drinking water and hygiene	Scope and results of raising awareness Status/Score: X	Y	Indicator not auditable The Strategy targets all groups; the AP only targeted educators, teachers, children, and parents
8. Setting hygiene requirements for swimming pools	Adopted regulations Status/Score: G	Y	Rules on minimum hygiene requirements for bathing water in swimming pools were adopted in 2015
9. Swimming areas, monitoring water quality and informing the public	Marked swimming areas and informative dashboards placed Status/Score: W	Y	Monitoring and public information was provided for municipal swimming pools and coastal water swimming areas

*AP* action plan, *APR* action plan rationale, *GIS* geographic information system, *NIJZ* National Institute of Public Health

^a^Indicators have been defined by the AP or are based on the APR

**Table 3:** Audit findings regarding priority Goal 3 (Slovenia 2012–2019)

Priority Goal 3: Disease prevention by improving indoor and outdoor air quality			
Activities planned for achieving the expected results—AP Reduction in exposure to particulate matter and other substances. Activity 1 (a–c) AQ monitoring and forecasting. Activity 2 Intersectoral policies that reduce indoor air pollution, including radon. Activities 3 and 4			
AP activities	Indicators and performance evaluation of the activities	Audit findings	
		Consistency	Additional comments (according to Table 1)
1. Encouraging municipalities to (a) Plan non-commercial infrastructure away from busy roads (b) Integrate sustainable mobility solutions into spatial policy, and (c) Introduce greater energy efficiency and RES Stricter control of individual household biomass combustion (and prevention of waste combustion)	1.1 Adopt and implement guidelines for considering human health in spatial planning Status/Score: W 1.2 Share of people living near busy roads Status/Score: O 1.3 Expand bicycle network Status/Score: O 1.4 Increased use of public transport Status/Score: G 1.5 Energy efficiency, household energy use and use of RES Status/Score: W 1.6 Control over household combustion systems Status/Score: W	P (e.g. unclear roles and obligations of municipalities)	1.1 The Spatial Planning Act of 2018 broadly defines health protection directions for municipal spatial planning (no direct rules) and encourages municipalities to provide the connectivity of green and built open spaces within and outside settlements 1.2, 1.3 No consistent and accessible data 1.4 Volume of public transport (rail and road) has increased from 39 to 41 million passengers; car use has also increased 1.5 Electric energy use has increased and so have the shares of RES and energy efficiency. Energy policy is set at the national level 1.6 No effective control over the quality of household wood combustion systems or the amount/type of waste burnt in households
2. Upgrading AQ monitoring and forecasting systems	2.1 Establish an air pollution forecasting system and a user-friendly web portal Status/Score: W 2.2 Number of measuring points and parameters Status/Score: W	Y	2.1 Implemented forecasting system and web portal. No data on the effectiveness regarding citizens’ health improvement 2.2 National AQ monitoring network expanded from 18 to 22 measuring points. No change in number of measured parameters
3. Linking health and environmental inspections	An established inter-ministerial working group Status/Score: W	N	Not among activities of the Strategy. No public information on the group’s establishment
4. Radon Monitoring: (a) Exposure at the national level (b) Recommendations on permissible concentrations in areas where children spend the most time (c) Remediation work on buildings, and (d) Measures to reduce radon concentrations	4.1 A radon atlas Status/Score: P 4.2 Annual measurements of radon concentrations at refurbished facilities Status/Score: O 4.3 Proportion of remediated buildings Status/Score: X 4.4 Use of materials and construction methods to prevent elevated radon concentrations Status/Score: X	N	4.1 Not consistent. Radon is discussed in another goal of the Strategy, not in AQ monitoring No radon atlas. A list of municipalities with higher potential of elevated radon levels is available 4.2 No data available 4.3 Indicator not auditable. No definition of ‘buildings in need of remediation’, no remediation specifications, etc 4.4 Indicator not auditable. No specifics on construction materials and methods, sectors for implementation, etc

*AP* action plan, *AQ* air quality, *RES* renewable energy sources

- G—Good performance of the activity (complete and quality), results documented and auditable
- W—Weak performance of the activity, results unclear/non-transparent or poorly documented
- O—Not observed or evaluated. Available information was not complete enough for thorough evaluation
- X—Not applicable: evaluation based on selected indicators is not applicable (sensible)
- Y—Consistent: full overall or specific consistency between the Strategy and AP
- N—Not consistent: Strategy and AP are not consistent
- P—Partial consistency between the Strategy and AP

## Results

Results for priority Goals 1 and 3 are presented in Tables 2 and 3 (Legal Information System 2015; Okorn 2016; National Institute of Public Health 2019, 2020a, b). Similar findings are available for priority Goals 2 and 4; however, they are not shown here due to space limitations.

## Discussion

Limited healthcare resources and related issues make evaluating the impact of public health interventions increasingly important (Mays and Smith 2011; Méndez and Osorio 2017; Bernet et al. 2018; Saeed et al. 2019). The need for more child and adolescent health research was emphasised in relation to the child and adolescent health strategy development (Dratva et al. 2018). The auditing of the Strategy and its AP provided a framework to encourage and facilitate continuous evaluation of the effectiveness of activities with a specific focus on the health of children and adolescents in relation to the environment. The activities of the AP were both preventive and curative and concerned environmental quality. Regarding adolescent health, however, they were strictly preventive, with no evidence for necessary interventions prior to the implementation of the Strategy. In this context, the AP activities aimed at improving environmental quality can yield a positive long-term health impact. The Strategy and AP are not fully consistent; the Strategy’s time span is from 2012 to 2020, but the AP’s activity plans cover 2015–2020. The AP includes some additional topics and activities, but it does not include some topics identified as important by the Strategy. It also fails to include some of the Strategy’s specific areas of interest, e.g. youth participation, new technology, etc.

Policy effectiveness (e.g. measured by expenditures, investment costs or timing) does not necessarily lead to success in terms of the policy’s original goals. However, the challenge when evaluating the effectiveness of public health efforts, especially in an environmental context, requires the development of appropriate metrics for evaluating health changes resulting from different policy approaches (Kingdon 1995; Brownson et al. 2010). This is one of the audit’s key findings. Only a few indicators (Tables 2, 3) demonstrate the AP activities’ good performance with well-documented results, while the majority show either weak performance or could not be evaluated due to poor or absent data. Several indicators defined by the AP are not fit for their intended purpose in terms of evaluating the effectiveness or success of actions (Table 2 indicators 2.2, 3, 4.1, 6, and 7; Table 3 indicators 4.3 and 4.4). Examples of such indicators are those related to drinking water quality and city air pollution associated with public transport. These indicators suffer from unclear goals and intended uses; as a result, it was not possible to evaluate their impact on health improvements. A number of indicators include ‘raising awareness’ and ‘informing the public’ without providing specifics about the events to be included in the evaluation, groups to be addressed, etc. Most of the activities and their indicators do not specifically target children or adolescents but rather focus on the entire population. This presents a barrier in the assessment of associations between environmental quality and specific child and adolescent health outcomes.

**Fig. 2:**
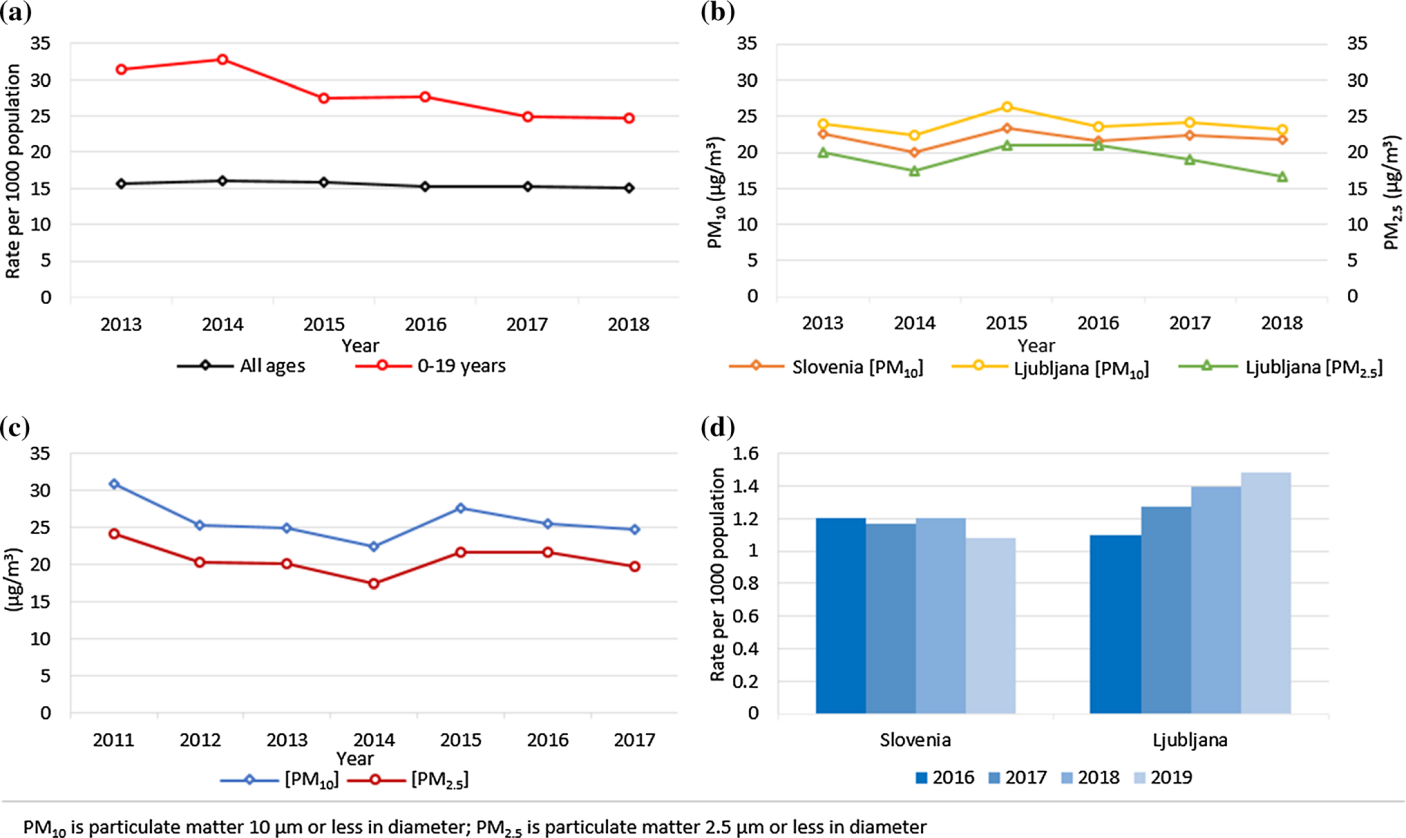
Air quality and adolescent health in Slovenia between 2013 and 2018. **a** Annual hospitalisations due to respiratory conditions by age group in Slovenia from 2013 to 2018; **b** concentrations of PM_10_ (Slovenia and Ljubljana) and PM_2.5_ (Ljubljana) from 2013 to 2018; **c** potential exposure of urban population to PM_10_ and PM_2.5_ air pollution in Slovenia from 2011 to 2017; **d** annual asthma-related hospitalisations in children and adolescents under 20 years of age in Slovenia and Ljubljana from 2016 to 2019

The auditing highlighted the issue of inconsistent and indirect associations between specific available environmental quality data and potential (assumed) exposure with specific health outcomes. This is illustrated by an example (Fig. 2), though several have been observed during the audit (National Institute of Public Health 2020a; SEA 2020). Figure 2 shows the issues in determining associations between air quality data and health outcomes (National Institute of Public Health 2020b, c; SEA 2019; Statistical Office of the Republic of Slovenia 2019). Levels of PM_10_ and PM_2.5_ were largely constant in the entire observed period (± 5 µg/m^3^ seasonal variations) (SEA 2019). That said, the hospitalisation of children and adolescents due to respiratory diseases decreased (National Institute of Public Health 2020b). In the city of Ljubljana, asthma-related hospitalisations increased by almost 35% from 2016 to 2019, while PM_10_ and PM_2.5_ concentrations stayed the same or even decreased (National Institute of Public Health 2020c). Changes in hospitalisation due to respiratory diseases could be explained by several reasons not directly associated with air quality, such as behavioural changes, the impact of influenza season, varying health data records in the health information system, different meteorological conditions, variations in sensitivity, etc. Another issue in analysing the data involves inconsistencies in their interpretation, as highlighted in Fig. 2c. The plot presents PM_10_ and PM_2.5_ concentrations, while the formal interpretation as provided by the data source defines them as ‘population exposure data’ (Statistical Office of the Republic of Slovenia 2019). Such inconsistencies hinder the process of evaluating the success of the Strategy.

In terms of the IWG’s expected versus actual work, we conclude that there could have been greater transparency, including in its reporting of the Strategy implementation and of goals achieved (based on publicly available information). Moreover, transparency regarding the participation of interested parties is not clear. Collaboration between sectors, NGOs or youth organisations is reported (Ministry of Health of the Republic of Slovenia 2015); however, no information on the effectiveness of such collaborations are available.

### Limitations

The audit was performed based on publicly available information. Additional data could improve the overall review of the Strategy and its impacts.

### Conclusions

There is no clear evidence that the Strategy has contributed to positive changes in child and adolescent health in Slovenia during the period 2012–2019. Therefore, proposals for future work are as follows:

- Monitoring policy implementation and its results is crucial, and metrics should be defined in detail along with policy.
- Environmental health indicators should be fit for their intended purposes.
- Effective intersectoral work is needed (e.g. a permanent body comprising involved sectors) and is crucial for successful public health interventions (Bjegovic-Mikanovic et al. 2018).
- Audits should be properly planned and systematically performed. They should be understood as an integral part of monitoring any policy implementation. In this view, no public policy is to be excluded from performance auditing; as has been observed recently, not even those of the WHO (Nature 2020).
- Re-auditing is vital; without undertaking re-audits regularly, there is no way of knowing whether the midcourse corrections that have been made have improved the situation.
- Attention should be paid to the current and forthcoming issues affecting the health of young people: sleeping and eating habits, economic migration, changes in family structure, drop in fertility rates, poverty, etc.
